# Structural and functional association of androgen receptor with telomeres in prostate cancer cells

**DOI:** 10.18632/aging.100524

**Published:** 2013-01-29

**Authors:** Junying Zhou, Michelle Richardson, Vidyavathi Reddy, Mani Menon, Evelyn R. Barrack, G. Prem-Veer Reddy, Sahn-Ho Kim

**Affiliations:** Vattikuti Urology Institute, Henry Ford Hospital, Detroit, MI 48202

**Keywords:** Androgen receptor, telomeres, fragile telomeres, Casodex, DNA damage, genomic instability, prostate cancer

## Abstract

Telomeres protect the ends of linear chromosomes from being recognized as damaged DNA, and telomere stability is required for genome stability. Here we demonstrate that telomere stability in androgen receptor (AR)-positive LNCaP human prostate cancer cells is dependent on AR and androgen, as AR inactivation by AR antagonist bicalutamide (Casodex), AR-knockdown, or androgen-depletion caused telomere dysfunction, and the effect of androgen-depletion or Casodex was blocked by the addition of androgen. Notably, neither actinomycin D nor cycloheximide blocked the DNA damage response to Casodex, indicating that the role of AR in telomere stability is independent of its role in transcription. We also demonstrate that AR is a component of telomeres, as AR-bound chromatin contains telomeric DNA, and telomeric chromatin contains AR. Importantly, AR inactivation by Casodex caused telomere aberrations, including multiple abnormal telomere signals, remindful of a fragile telomere phenotype that has been described previously to result from defective telomere DNA replication. We suggest that AR plays an important role in telomere stability and replication of telomere DNA in prostate cancer cells, and that AR inactivation-mediated telomere dysfunction may contribute to genomic instability and progression of prostate cancer cells.

## INTRODUCTION

Telomere DNA at the ends of chromosomes is double-stranded (DS) except for the single-stranded (SS) 3’-overhang [1]. This overhang would be recognized as damaged DNA, were it not for the presence of protein complexes [2, 3]. Thus, telomeres are DNA-protein structures that cap the ends of chromosomes and protect them from a DNA damage response that can lead to chromosome structural abnormalities or cell death [4-12]. Telomeres contain many different proteins that play a role in the maintenance of telomere stability; the best characterized are the six proteins (TRF1, TRF2, Rap1, TIN2, POT1 and TPP1) that comprise the complex known as shelterin [4]. TRF1 and TRF2 bind to DS telomere DNA, and POT1 binds to the SS 3’ overhang [13-16]; all other components of the telomere are the result of protein-protein interactions [4, 5]. Structural and functional stability of telomeres require not only the complex of shelterin proteins but also a growing number of non-shelterin proteins to ensure proper and timely repair and replication of telomere DNA. Many of these non-shelterin proteins are only transiently associated with telomeres, being recruited for their role in DNA repair (Ku70/80, XPF/ERCC1, Apollo) [17-19], DNA damage signaling (Mre11 complex, 9-1-1 complex, RAD51, BRCA2) [20-23], DNA replication (CTC1-STN1-TEN1 [CST] complex, Origin Recognition Complex [ORC], RecQ helicase) [24-26] or chromatin structure (HP1 proteins) [27]. By contrast, shelterin is present at telomeres throughout the cell cycle [5]. Telomeres turn over once per cell cycle, during S phase. Telomere disassembly and reassembly are closely coordinated with the replication of telomere DNA, in order to prevent chromosome ends from being detected as lesions and triggering a DNA damage response signal. Telomere assembly entails the recruitment of proteins involved in DNA repair, replication and recombination [28], suggesting that these non-shelterin proteins are important components of telomere structure and function.

Functional telomeres are essential for maintaining genome integrity; telomere shortening resulting from telomere dysfunction leads to genomic instability, which is a common cause and hallmark of cancer [29-31]. The enzyme telomerase [32], and occasionally the telomerase-independent alternative lengthening of telomeres (ALT) pathway [33], play an important role in preventing progressive shortening of telomeres during cell division. However, telomere dysfunction and telomere shortening can result from single strand breaks in telomere DNA caused by oxidative DNA damage [34] or from defective shelterin or non-shelterin proteins associated with telomere DNA replication [23, 35-37]. Telomere shortening is an early event in prostate carcinogenesis [38, 39] and it is associated with genomic instability in prostate tumor tissues [40]. Interestingly, genomic instability is also associated with the progression of prostate cancer from androgen-dependence to castration resistance [41]. While the telomere shortening in early stages of disease development can be attributed to the proliferation of cells in the absence of telomerase, the molecular basis for genomic instability in later stages of prostate cancer when telomerase is likely to be reactivated remains a mystery [42, 43].

We reported previously the surprising observation that the specific androgen receptor (AR) antagonist bicalutamide (Casodex) causes telomere dysfunction and that AR interacts with shelterin proteins in AR-positive LNCaP prostate cancer cells [44], suggesting a role of AR in telomere stability and function. In the present study we tested whether a) Casodex-induced telomere dysfunction is indeed mediated by AR, b) AR interaction with shelterin proteins occurs at telomeres, and c) AR inactivation causes telomere abnormalities in prostate cancer cells. Our studies demonstrated for the first time that AR is involved in telomere function since androgen-deprivation or AR-siRNA caused telomere dysfunction, that AR is associated with telomere chromatin as revealed by a protocol to isolate telomeric chromatin, and that AR inactivation causes telomere breakage and sister chromatid telomere fusion as determined by fluorescence *in situ* hybridization (FISH) analysis. These latter events are reminiscent of those that occur in cells with defective shelterin and non-shelterin proteins at telomeres [23, 36]. We conclude from our observations that AR is a structural component of telomeres and that AR inactivation causes telomere dysfunction through a mechanism that is independent of its role as a transcription factor in regulating the expression of AR-target genes.

## RESULTS

### Casodex induces telomere dysfunction via its effect on AR

We previously reported that the specific AR antagonist Casodex, added at a concentration of 100 μM to cells in 10% serum-containing medium (complete), causes a TIF response in AR-positive LNCaP cells [44]. Contrary to the relative effectiveness of 10 μM Casodex in blocking AR activity in steroid-depleted medium [45-47], a higher concentration of Casodex, 80-100 μM, is needed to similarly inhibit AR activity in complete medium (S. Murthy, U. Bai and G.P. Reddy; manuscript in preparation; [47]), likely because of a high concentration of steroids in fetal bovine serum, many of which can bind to the mutated AR in LNCaP cells. Notably, the steady-state serum level of Casodex in prostate cancer patients treated with 150 mg Casodex/day is reported to be approximately 27 μg/ml (90 μM) [48, 49]; this indicates that the concentration of Casodex we use *in vitro* is pharmacologically relevant *in vivo*.

**Figure 1:**
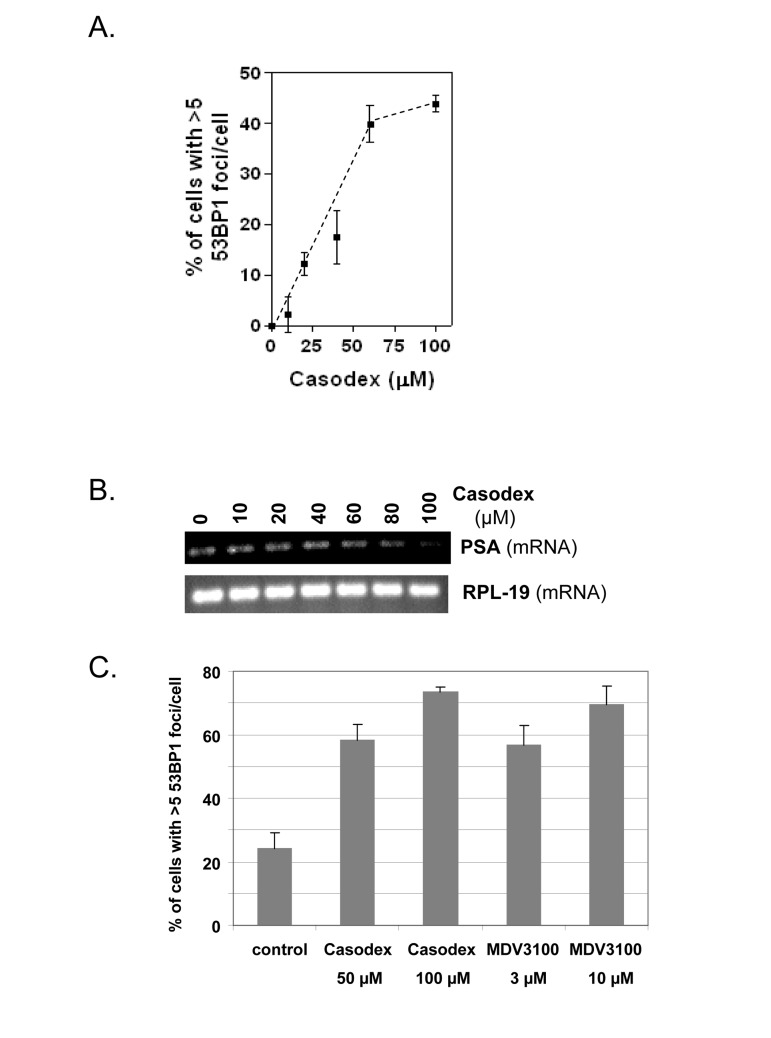
AR antagonists cause a TIF response (**A**), LNCaP cells treated with or without Casodex for 24 hrs were analyzed for the percentage of cells with >5 53BP1 foci/cell; data are expressed relative to untreated control cells. (**B**), PSA mRNA levels in cells from (**A**) were measured by RT-PCR. RPL-19 is a loading control. (**C**), LNCaP cells were treated with or without Casodex (50 μM, 100 μM) or MDV 3100 (3 μM, 10 μM) for 24 hr, then evaluated for the percentage of cells with >5 53BP1 foci/cell.

Fig. 1A shows dose-dependence of the TIF response to Casodex (IC_50_ ~40 μM), and Fig. 1B shows that the inhibition of PSA expression by Casodex has a similar dose-response. Notably, the IC_50_ for Casodex-mediated inhibition of PSA expression is ~40 μM in FBS or CSS (S. Murthy, U. Bai and G.P. Reddy; manuscript in preparation). Thus, the TIF response to Casodex is associated with AR inactivation; this implicates a role for AR in telomere stability, but does not address the question whether AR transcriptional activity is required for this role.

Notably, the TIF response to AR inactivation is not unique to Casodex. Fig. 1C shows that MDV3100, a newly developed AR antagonist with 5-8 fold higher affinity for AR, is indeed more potent than Casodex in inducing a TIF response, as 3 μM MDV3100 had the same effect as 50 μM Casodex, and 10 μM MDV3100 had the same effect as 100 μM Casodex.

**Figure 2:**
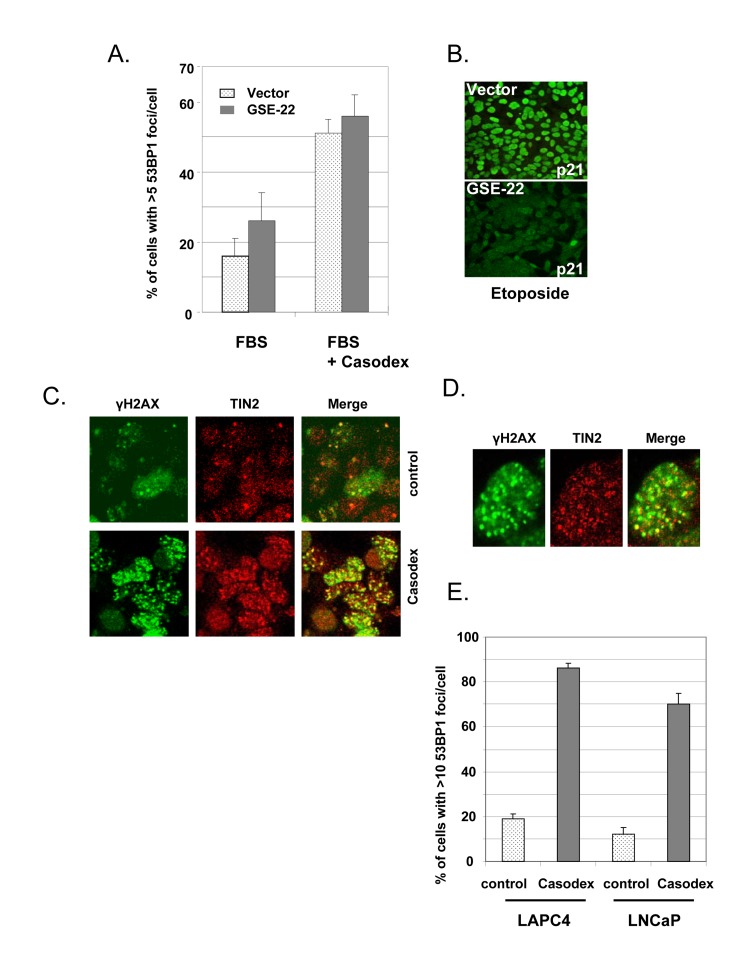
Casodex-induced TIF response is independent of p53 and AR mutation status (**A**, **B**), Casodex-induced TIF response is independent of p53. LNCaP cells were infected with a dominant-negative p53 (GSE-22) or control retroviral expression vector, and selected for 2-3 d. Cells were then treated (A) with or without 100 μM Casodex for 24 hr, and then analyzed for 53BP1 foci [44], or (B) with or without 10 μg/ml etoposide for 24 hr, then fixed and stained with p21 antibody. (C-E), Casodex induces a TIF response in AR-positive LAPC4 cells with wild-type AR. (**C**), LAPC4 cells were treated with or without 100 μM Casodex for 24 hr and then co-immunostained with antibodies to γH2AX (green) and TIN2 (red). Colocalization of γH2AX and TIN2 is shown in the ‘merge’ panel. (**D**), Higher magnification images of a representative Casodex-treated LAPC4 cell stained with antibodies to γH2AX and TIN2. (**E**), LAPC4 cells and LNCaP cells were treated with and without 100 μM Casodex for 24 hr, then evaluated for the percentage of cells with >10 53BP1 foci/cell. Bars represent the mean ± standard deviation.

Since the only known target of Casodex is the AR, we interpreted the failure of Casodex to induce a TIF response in AR-negative PC3 prostate cancer cells as due to the absence of AR [44]. However, since the p53 status also differs between LNCaP (wild-type p53) *vs*. PC3 cells (inactive mutant p53) [50], we sought to rule out the possibility that the TIF response is mediated by or requires wild-type p53. Therefore, p53 was inactivated in LNCaP cells by expressing a dominant-negative p53 element (GSE-22, described by [51]). Whereas etoposide treatment of control vector-transfected LNCaP cells induced the expression of p21^Cip1^ (a p53 target gene) (Fig. 2B, upper panel), GSE-22 prevented this effect of etoposide (Fig. 2B, lower panel), thereby demonstrating effective inactivation of p53. Notably, inactivation of p53 did not prevent the TIF response to Casodex (Fig. 2A); therefore, the TIF response to Casodex in LNCaP cells is independent of and not mediated by p53.

Since the LNCaP cell AR is mutated [52], we tested the effect of Casodex on LAPC4 prostate cancer cells, which have wild-type AR [53]. As shown in Fig. 2C, we observed a dramatic increase in the number of immunofluorescent γH2AX foci in Casodex-treated LAPC4 cells as compared to controls. These γH2AX foci in Casodex-treated cells colocalized with TIN2 (Fig. 2C and 2D), indicating the presence of dysfunctional telomeres. The TIF response of LAPC4 cells to Casodex treatment was similar to that of LNCaP cells (Fig. 2E). Therefore, Casodex induces a DNA damage response in prostate cancer cells with wild-type AR (LAPC4 cells) or mutant AR (LNCaP cells).

### Androgen is required for telomere stability

**Figure 3:**
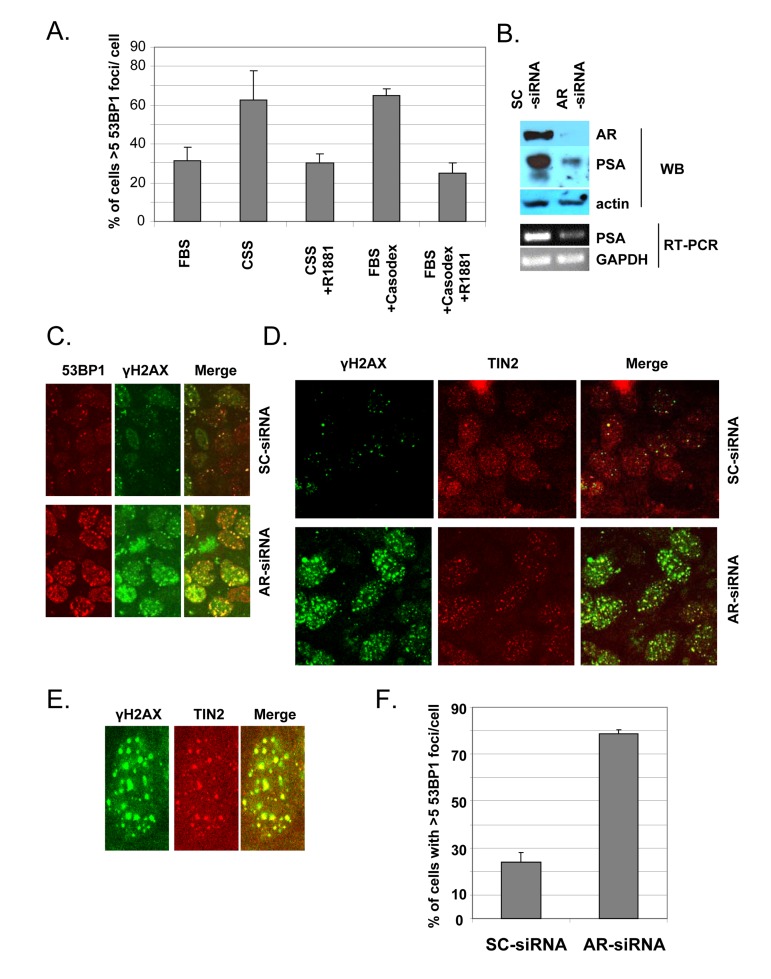
Androgen-depletion or AR knockdown triggers a TIF response (**A**), Androgen -depletion triggers a TIF response. Exponentially growing LNCaP cells in complete medium were either left untreated (bar labeled FBS), or were treated with 100 μM Casodex (FBS+Casodex) or Casodex + 10 nM R1881, for 24 hr. To determine the effect of androgen depletion, LNCaP cells growing in complete medium (FBS) were switched to steroid-free medium (CSS, phenol red-free medium containing 10% charcoal-stripped serum) or CSS medium + 10 nM R1881, and incubated for 24 hr. Data are presented as the percentage of cells with >5 53BP1 foci/cell. Bars represent the mean ± standard deviation. (**B, C, D**), AR knockdown triggers a TIF response. Exponentially growing LNCaP cells were treated with AR-siRNA (AR) or scrambled (SC)-siRNA for 48 hr. (**B**), Cell extracts were subjected to Western blot to confirm knockdown of AR protein and PSA, an AR target gene. Actin was used as a loading control. Total RNA was extracted from siRNA-treated cells using Trizol (Invitrogen) and RT-PCR was performed as described previously [44] to measure mRNA levels using sequence specific primers for GAPDH and PSA as described previously [86]. (**C**), Cells were stained with antibodies to 53BP1 (red) and γH2AX (green). (**D**), Cells were stained with antibodies to γH2AX (green) and TIN2 (red) in order to evaluate the TIF response to AR knockdown. (**E**), Higher magnification images of a representative AR-siRNA-treated cell stained with antibodies to γH2AX and TIN2. (**F**), Quantitation of dysfunctional telomeres following AR knockdown or control SC-siRNA, based on the percentage of cells with >5 53BP1 foci/cell.

Although Casodex and MDV3100 are known as specific AR antagonists [54-57], and the TIF response appears to be due to AR inactivation (Fig. 1B), we tested the effect of androgen depletion as an alternative approach to inactivate AR. Since Casodex binding to AR causes nuclear accumulation of AR and binding to target genes, though without activating them [47], we considered the possibility that the TIF response to Casodex might be ligand-independent, in which case androgen depletion might not cause a TIF response. As shown in Fig. 3, switching cells from complete medium with serum to medium with charcoal-stripped serum caused a TIF response, and this effect was abrogated by the addition of R1881, a synthetic androgen. Therefore, androgen is required for telomere stability. The TIF response to Casodex also was abrogated by the addition of R1881 (Fig. 3), confirming that the effect of Casodex was due to AR antagonism and not a nonspecific toxic effect.

### AR is required for telomere stability

In order to provide direct evidence for a role of AR in telomere stability, we used RNA interference with AR-siRNA to inhibit AR production in LNCaP cells. Transfection of cells with AR-siRNA reduced the AR protein level by ~90%, compared to control transfection with scrambled (SC)-siRNA (Fig. 3B), and the decrease in AR was associated with a concomitant decrease in AR-dependent PSA protein and mRNA (Fig. 3B). As illustrated in Fig. 3C, AR-siRNA treatment caused a dramatic increase in the number of immunofluorescent γH2AX or 53BP1 foci, compared to SC-siRNA controls; this indicates that AR knockdown caused a DNA damage response. The colocalization of 53BP1 foci and γH2AX foci (Fig. 3C) indicates that γH2AX and 53BP1 represent the same DNA damage foci. The colocalization of γH2AX with TIN2 in AR-siRNA-treated cells (Figs. 3D and E) indicates that the DNA damage response is at telomeres. Quantitation of this response (Fig. 3F) indicates that AR knockdown with AR-siRNA caused a dramatic TIF response, compared to control treatment with SC-siRNA.

### AR transcriptional activity is not required for telomere stability

**Figure 4:**
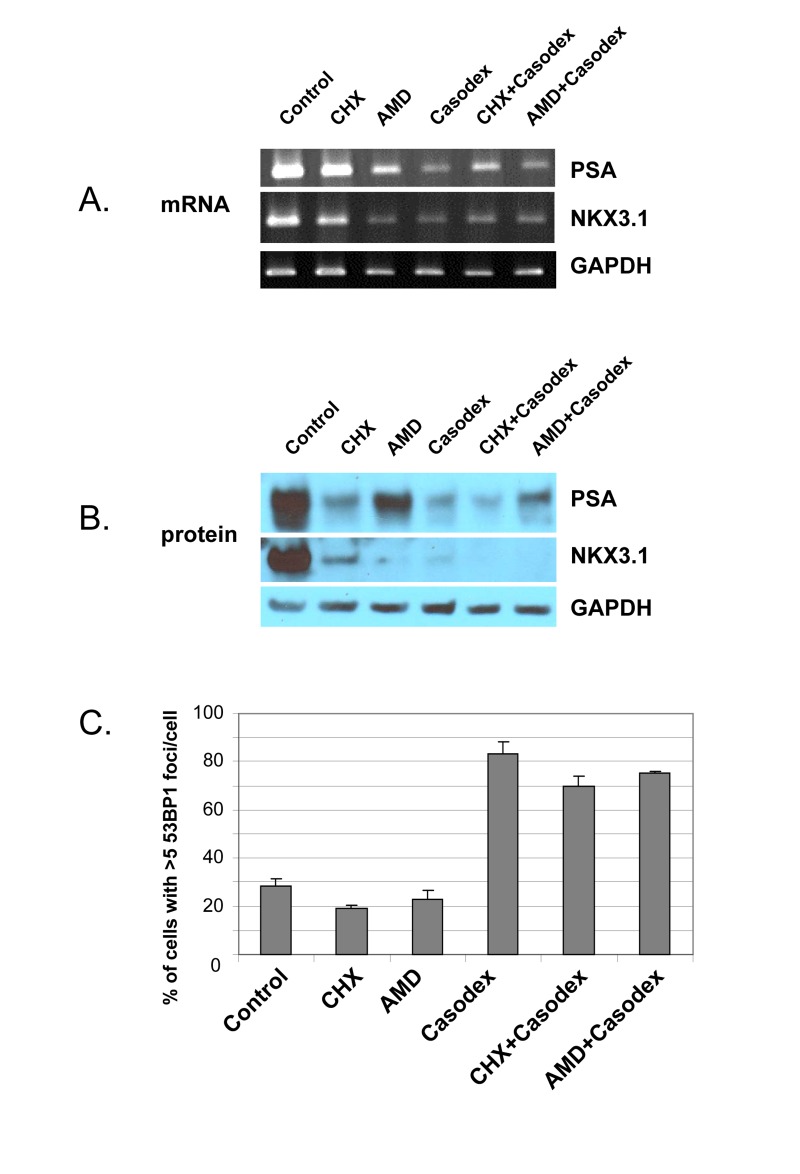
Casodex-induced TIF response in LNCaP cells is not due to inhibition of transcriptional activity or protein translation LNCaP cells were treated with cycloheximide (CHX, 20 μg/ml), actinomycin D (AMD, 0. 5 μg/ml), Casodex (100 μM), Casodex+CHX, or Casodex+AMD for 24 hr. (**A**), mRNA levels were assayed by RT-PCR; (**B**), protein levels were assayed by Western blot analysis; (**C**), cells were evaluated for a TIF response, based on the percentage of cells with >5 53BP1 foci/cell.

Since the TIF response to Casodex is associated with inhibition of AR transcriptional activity (Fig. 1), we considered the possibility that the TIF response was due to the inhibition of AR transcriptional activity. Therefore, we treated cells with the general transcription inhibitor actinomycin D or the translation inhibitor cycloheximide in order to determine the role of *de novo* biosynthesis in telomere stability (Fig. 4). Actinomycin D decreased the mRNA level (Fig. 4A), and cycloheximide decreased the protein level (Fig. 4B), of AR target genes PSA and NKX3.1, but neither actinomycin D nor cycloheximide caused a TIF response (Fig. 4C). Thus, inhibition of *de novo* biosynthesis of mRNA and protein was not sufficient to induce a TIF response.

However, these data do not rule out the possibility that the TIF response to Casodex is due to inhibition of AR transcriptional activity, as AR transcriptional activity increases the expression of some genes, and *decreases* the expression of other genes [58, 59]; therefore, inhibition of AR transcriptional activity by Casodex would be expected to *increase* the expression of genes down-regulated by AR. Therefore, in order to determine whether the TIF response to Casodex is mediated by up-regulation of such genes, we co-treated cells with Casodex and actinomycin D to inhibit the expression of genes potentially up-regulated by Casodex, or with Casodex and cycloheximide (Fig. 4C). Notably, neither actinomycin D nor cycloheximide blocked the TIF response to Casodex (Fig. 4C); thus, the TIF response to Casodex is not mediated by an effect on AR transcriptional activity, and the role of AR in telomere stability does not require AR transcriptional activity.

### AR is associated with telomeric chromatin

**Figure 5:**
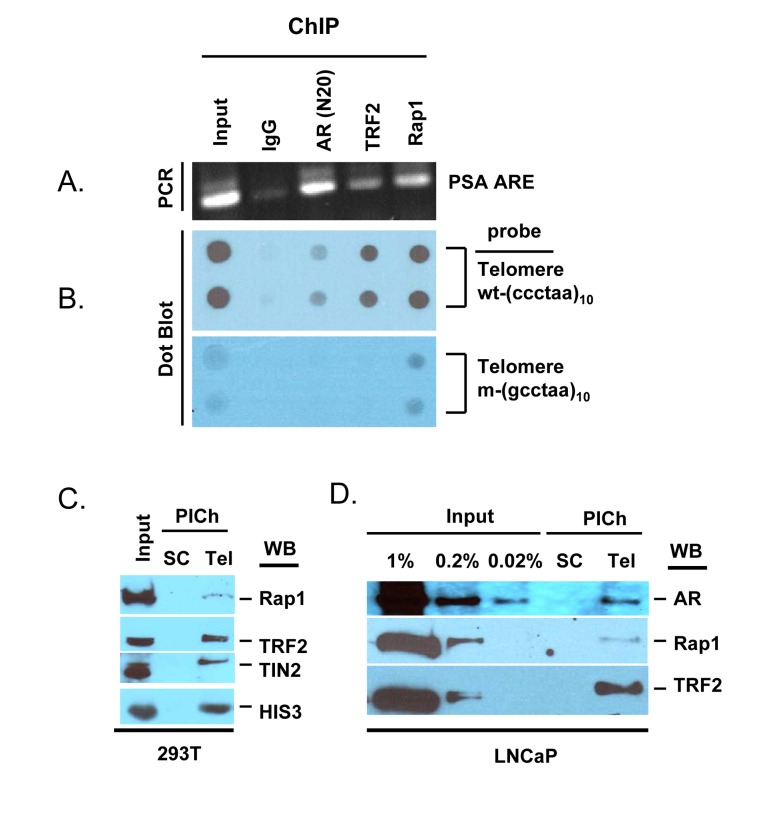
AR is a component of telomeres (**A, B**), AR-ChIP contains telomere DNA. LNCaP cell chromatin was subjected to immunoprecipitation with antibodies to AR, TRF2, Rap1, or normal IgG (negative control). (**A**), DNA was purified from the ChIP and analyzed for the presence of AR binding sequences in the PSA gene (ARE III in PSA promoter/enhancer) [91]. (**B**), DNA purified from ChIP was loaded onto a nylon membrane using a dot blot apparatus and probed with a DIG-labeled telomere DNA repeat [wt-(ccctaa)_10_] or mutant DNA repeat [m-(gcctaa)_10_]. Input DNA represents 10% of total DNA, before immunoprecipitation. (**C, D**), Telomeric chromatin contains AR. The PICh protocol was used to isolate telomeric chromatin from total chromatin of 293T cells (**C**) or LNCaP cells (**D**), using a telomere-specific probe (Tel) or a scrambled sequence probe (SC). Isolates were analyzed by Western blot (WB) for the presence of Rap1, TRF2, TIN2, HIS3, or AR. Input (%) represents starting material.

Co-localization of AR and TIN2 (a component of shelterin in telomeres) in untreated LNCaP cells, and co-immunoprecipitation of AR with TRF1 and TRF2 (telomere DNA binding proteins), indicate the presence of a subset of AR in telomeres [44]. In order to provide further evidence that AR is a component of telomeres, we prepared chromatin from formaldehyde-fixed LNCaP cells, used AR antibody to immunoprecipitate AR-bound chromatin (AR-ChIP), and probed this fraction for the presence of telomere DNA. As a positive control to confirm that AR-ChIP contains AR-bound chromatin, we demonstrated the presence of PSA gene androgen response elements (ARE) (Fig. 5A). Interestingly, Rap1-ChIP and, to a lesser extent, TRF2-ChIP also contained PSA ARE (Fig. 5A); this is not surprising, in light of reports that Rap1 and TRF2, besides being components of shelterin at telomeres, may also be associated with transcribed genes [60-63].

Notably, telomere DNA was detected in AR-ChIP, based on the use of a digoxigenin-labeled 10-mer telomere repeat; by contrast, a mutated DNA repeat did not yield a signal (Fig. 5B). Thus, AR appears to be physically associated with telomeric chromatin in LNCaP cells. As positive controls, we demonstrated the presence of telomere DNA in TRF2-ChIP and Rap1-ChIP, consistent with the presence of these shelterin components in telomeres, whereas the absence of telomere DNA in control IgG-ChIP served as a negative control (Fig. 5B). The telomere DNA signal in AR-ChIP was substantially lower than that in TRF2-ChIP or Rap1-ChIP (Fig. 5B), suggesting AR association with a subset of telomeres.

Having demonstrated the presence of telomere DNA in AR-ChIP, we sought to demonstrate the presence of AR in telomeric chromatin. A novel protocol to isolate telomeric chromatin (referred to as the PICh protocol) [64] has been described recently; it is based on the use of a biotinylated telomere DNA repeat as a probe to hybridize, under stringent conditions (70°C), with endogenous telomere DNA in chromatin fragments of ~600 bp length; streptavidin-labeled beads are then used to pull down the telomere DNA with its associated proteins [64]. The stringent features of this protocol minimize the isolation of non-telomeric chromatin, and a biotin-labeled, scrambled DNA sequence not found in the genome is used as a negative control in this approach [64]. We used this approach to isolate telomeric chromatin and probed it for the presence of AR (Fig. 5C-D). Using 293T cells to optimize the PICh protocol, we demonstrated that the telomere probe pulled down chromatin that contained shelterin components, based on immunoblot analysis with anti-bodies to Rap1, TRF2, and TIN2, whereas the control scrambled probe did not (Fig. 5C). Thus, it appears that the telomere probe can be used to enrich for telomeres. This fraction also contained histone 3 (Fig. 5C), as telomeric chromatin contains nucleosomes [64, 65]. We then used this protocol to isolate telomeres from LNCaP cells; a subset of AR in total chromatin was present in the fraction (as were TRF2 and Rap1) that was pulled down with the telomere-specific probe, but not in the fraction pulled down with the scrambled probe (Fig. 5D). This indicates that a subset of AR is associated with telomeric chromatin in LNCaP cells.

### Casodex induces the formation of fragile telomeres

**Figure 6:**
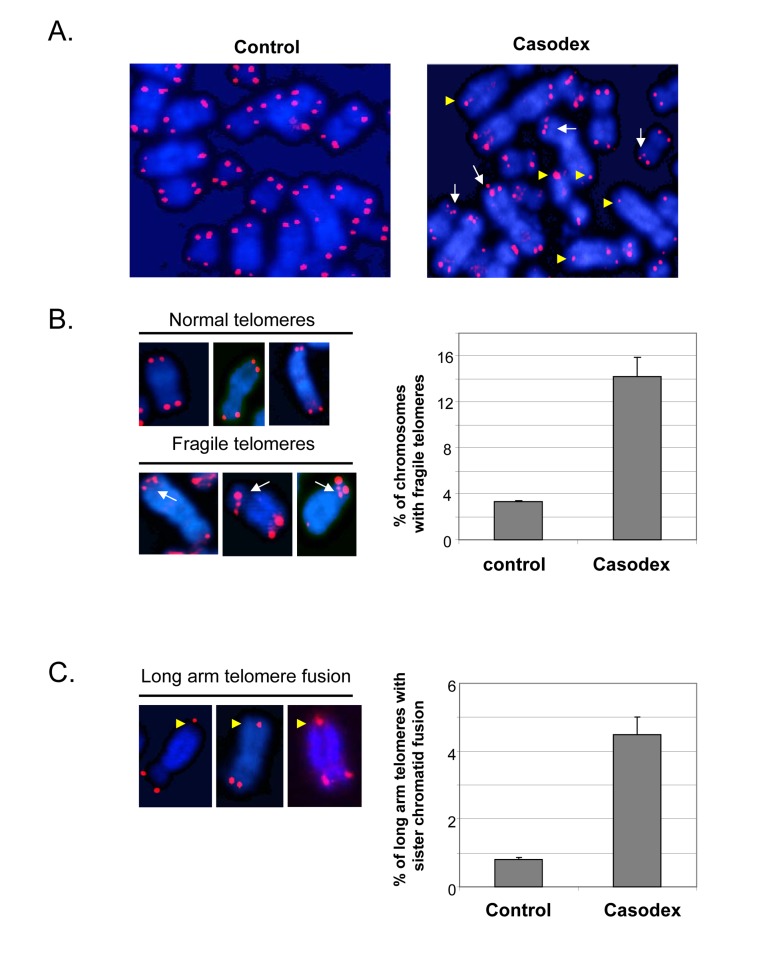
Casodex treatment causes fragile telomeres and sister chromatid fusion in LNCaP cells Metaphase spreads were prepared from LNCaP cells that had been treated without (control) or with Casodex for 24 hr, and then subjected to telomere-FISH. (**A**), Microscopic images showing representative metaphase spreads from control untreated and Casodex-treated LNCaP cells. Telomere-FISH labels telomeres red. DAPI counterstaining (blue) was used to visualize DNA. White arrows indicate chromosomes with multiple telomere signals, referred to as fragile telomeres. Yellow arrowheads indicate long arm sister telomere fusion. (**B**), Higher magnification images illustrate examples of normal telomeres and fragile telomeres of chromosomes from Casodex-treated LNCaP cells (left panel). Metaphase spreads were scored for the percentage of chromosomes with multiple telomere signals (fragile telomeres) (right panel). We evaluated 2095 chromosomes of control untreated cells, and 1390 chromosomes of Casodex-treated cells. Bars represent the mean ± standard deviation. (**C**), Higher magnification images illustrate examples of long arm sister chromatid fusion in Casodex-treated LNCaP cells (left panel). Metaphase spreads were scored for the percentage of chromosomes with long arm sister chromatid fusion (right panel). We evaluated 2072 chromosomes of control untreated cells, and 1164 chromosomes of Casodex-treated cells. Bars represent the mean ± standard deviation.

Since telomere dysfunction can lead to chromosome structural abnormalities, such as telomere end-to-end fusion [5], we investigated the consequence of telomere dysfunction caused by Casodex. Metaphase chromosome spreads were prepared from untreated and Casodex-treated LNCaP cells, and probed for the presence of telomere aberrations using fluorescence in situ hybridization of a labeled telomere probe (telomere-FISH) (Fig. 6). The telomere-FISH signal at individual chromatid ends is normally represented as a single signal with an intensity that is roughly equal to that of the sister chromatid end; this was the predominant pattern seen in control LNCaP cell metaphase spreads (Fig. 6A, left panel). A strikingly different pattern of telomere-FISH signals was seen in the metaphase spreads of Casodex-treated cells (Fig. 6A, right panel). Several chromosomes in Casodex-treated cells had multiple telomere signals (Fig. 6A right panel, white arrows; higher magnification shown in Fig. 6B, left panel). Of chromosomes with clearly discernible telomere signals, about 14% had multiple signals in Casodex-treated cells, compared to about 3% in control cells (Fig. 6B right panel).

The metaphase spreads of Casodex-treated cells (Fig. 6A right panel) also show some chromosomes with a single telomere signal, instead of two, one for each chromatid end. A single telomere signal at the end of a chromosome may represent the juxtaposition of two chromatid ends or the fusion of two sister chromatid telomeres; it is not possible to distinguish between these possibilities when the single signal is seen on the short arm of the chromosome. Therefore, we did not quantitate single signals at the short arm end of a chromosome. By contrast, a single telomere signal (instead of 2 signals) on the long arm of a chromosome with clearly separated long arms is likely to represent telomere fusion [yellow arrowheads in Fig. 6A and Fig. 6C (left panel)] [8, 10]; therefore, we quantitated the percentage of chromosomes with long arm sister telomere fusion (Fig. 6C). This phenotype was more common in Casodex-treated cells (Casodex, 4.5%; control, 0.8%).

Notably, a similar pattern of multiple telomere signals and sister telomere association in the long arms of telomeres has been found following knockdown of shelterin component TRF1, which leads to inhibition of telomere DNA replication and subsequently, telomere DNA breakage [10, 36]; the pattern of multiple telomere DNA signals is referred to as fragile telomeres [10]. It is known that telomeres are difficult to replicate because their repetitive arrays of guanosine-rich DNA sequences can form G quadruplexes that hamper progression of the DNA replication machinery [10, 66]. BLM, a TRF1-associated protein, and RTel have also been shown to play a role in telomere DNA replication [66], and their knockdown also causes an increase in fragile telomeres. Thus, our data suggest that the role of AR in telomere stability may occur via a role in telomere DNA replication.

The metaphase chromosomes of both Casodex-treated and control cells (Fig. 6A) also show chromatids with a weak telomere signal or no detectable telomere signal. Because the intensity of a telomere signal is proportional to telomere length, a weak or absent telomere signal may represent a shortened telomere, relative to other telomeres. Interestingly, telomere length of individual human chromosomes is heterogeneous [67], and the shortened telomeres of human cancer cells undergo dynamic shortening and lengthening [35]. Notably, the presence of weak or undetectable telomere signals in untreated human cancer cells has not been previously discussed.

## DISCUSSION

Telomeres and the proteins that protect them are essential for genome stability, as dysfunctional telomeres that cause telomere loss or telomere fusion result in abnormal chromosome segregation during mitosis. Thus, shelterin proteins and a growing number of accessory proteins help to maintain genome stability through their role in telomere stability and telomere DNA replication [5, 68]. We have shown that AR is a component of telomeric chromatin, and that AR inactivation causes dysfunctional telomeres, telomere breakage, and sister chromatid telomere fusion. Since telomere breakage and sister chromatid telomere fusion can contribute to genome instability [69], and since the progression of prostate cancer is associated with an accumulation of genetic changes [70], we propose that telomere aberrations observed in androgen-deprived or Casodex-treated (i.e. AR inactivated) cells may contribute to genetic instability, fueling the progression of prostate cancer. To the best of our knowledge, AR is the first steroid receptor shown to interact with telomeres and to be essential for telomere stability in prostate cancer cells.

A role of AR in telomere stability is based on our observations that AR inactivation by treatment with antiandrogen (Casodex or MDV3100) (Fig. 1C), androgen depletion (Fig. 3A), or AR knockdown (Fig. 3B-F) induced the formation of telomere dysfunction-induced foci (TIF), referred to as a TIF response. A role of AR in telomere stability is not unique to LNCaP cells that express mutant AR [52], as AR inactivation in LAPC4 cells that express wild-type AR [53] caused a similar TIF response (Fig. 2C-E). Although the TIF response to Casodex is associated with inhibition of AR transcriptional activity, as measured by a decrease in PSA mRNA level (Fig.1), inhibition of *de novo* RNA synthesis by actinomycin D or inhibition of *de novo* protein synthesis by cycloheximide did not cause a TIF response and did not block the TIF response to Casodex (Fig. 4). Thus, the role of AR in telomere stability cannot be explained by a transcriptional role of AR. Although the role of AR in survival and proliferation of prostate cancer cells is usually attributed to its role as a transcription factor [71, 72], there is increasing evidence for a non-transcriptional role of AR in a variety of cellular processes. For example, AR plays a role in: (a) activation of the Src-ERK pathway in the cytoplasm, independent of AR binding to nuclear DNA [73]; (b) DNA repair, through its interaction with Ku70 and Ku80, which bind to DNA double strand breaks [74]; (c) progression of cells from G_1_ to S phase, through its interaction with cell cycle regulatory proteins and enzymes required for initiation of DNA synthesis [75, 76]; and (d) recombination, by recruiting activation-induced cytidine deaminase (AID) and ORF2 endonuclease to promote DNA double strand breaks at translocation foci [77]. We propose that the role of AR in telomere stability also is independent of its transcriptional activity, and instead occurs through its interaction with shelterin proteins or shelterin-associated proteins in telomeric chromatin.

As expected, only a fraction of the total AR, TRF2 or Rap1 recovered with total chromatin (Fig. 5D, Input) was associated with telomeric chromatin (Fig. 5D, PICh). This likely reflects, at least in part, incomplete recovery of telomeric chromatin, and the known association of shelterin proteins, such as TRF2 and Rap1, with telomeric chromatin as well as with non-telomeric chromatin, where they play a role in regulating transcription, repair, and recombination [60-63]. In analogous fashion, AR associates with specific genes, where its function as a transcription factor regulates gene expression, and AR also associates with telomeric chromatin, where its function is independent of its role as a transcription factor (Figs. 4, 5, 6).

Our observation that Casodex induced telomere breakage and sister telomere fusion (Fig. 6) suggests a role of AR in telomere DNA replication. DNA breakage results from partial inhibition of DNA synthesis and activation of the DNA damage response to stalled replication at fragile sites in chromatin [78, 79]. Fragile sites are secondary structures in chromatin that challenge replication, particularly under conditions in which the efficiency of the DNA replication machinery is compromised [79]. The hairpin G-quadruplex DNA structures formed by TTAGGG repeats in telomere DNA represent such fragile sites [79, 80]. The efficient replication of telomere DNA requires TRF1; TRF1 deletion leads to the formation of multitelomeric signals (MTS) that are the result of telomere breakage [10, 36, 81]. In light of the physical association of AR with telomeric chromatin and its associated shelterin components TRF1, TRF2 and TIN2 ([44] and Fig. 5), it is conceivable that AR inactivation may impact the function of TRF1 and, thereby, cause telomere breakage during telomere DNA replication. Alternatively, since AR is associated with the DNA replication machinery ([75]; S. Murthy, U. Bai and G.P. Reddy; manuscript in preparation), and since telomere DNA replication utilizes the DNA replication machinery [82], it is conceivable that AR inactivation may compromise the efficiency of the DNA replication machinery during the replication of fragile sites in telomeres. Additional studies are needed to distinguish between these possibilities. In any event, it is evident from our observations that AR inactivation causes telomere breakage and sister telomere fusion, resembling what occurs in mouse embryo fibroblasts conditionally deleted for TRF1, which is required for telomere DNA replication, or treated with aphidicolin, which inhibits DNA polymerase activity required for telomere DNA replication [10, 36].

Androgen deprivation by surgical or pharmacological means is frontline therapy for the treatment of locally advanced or metastatic prostate cancer. Although this therapy is highly effective initially in controlling the growth of prostate cancer, the disease eventually becomes castration resistant. The mechanism behind this irreversible progression of prostate cancer remains largely unknown. Interestingly, the generation of castration resistant variants from prostate tumor xenografts subjected to androgen deprivation is associated with an increased level of genome instability [41]. This is consistent with our proposal that androgen and AR play a role in the maintenance of genome stability. Thus, AR inactivation by androgen deprivation or antiandrogen treatment, which leads to telomere dysfunction and telomere aberrations that include telomere breakage and sister chromatid telomere fusion, may contribute to castration resistant growth of prostate cancer cells through a mechanism involving telomere instability and subsequent genome instability. Future studies focused on identifying how AR interacts with other proteins in thetelomere may provide insight into how AR regulates telomere function; this could lead to an approach to avert genome instability and development of castration-resistant prostate cancer in patients subjected to androgen ablation.

## METHODS

### Cell culture

LNCaP (ATCC), LAPC4 (a generous gift from Drs. Robert Reiter and Charles Sawyers) or 293T cells (ATCC) were grown in RPMI (Gibco BRL), Iscove (Gibco BRL), or D-MEM medium (Gibco BRL), respectively, each containing 10% fetal bovine serum (FBS), 2.5 mM glutamine, 100 μg/ml streptomycin and 100 U/ml penicillin (complete medium). Exponentially growing cells, in complete medium, were treated with or without 20 μg/ml cycloheximide (Sigma), 0.5 μg/ml actinomycin D (Sigma), 100 μM bicalutamide (Casodex from LKT Laboratories, MN) or 10 μM MDV3100 (Selleckchem, TX). Steroid-depleted medium was phenol red-free RPMI medium (Gibco BRL) containing 10% charcoal-stripped serum (CSS, from Gibco BRL), 2.5 mM glutamine, 100 μg/ml streptomycin and 100 U/ml penicillin.

### Measurement of a TIF response

Telomere disruption leads to a DNA damage response, which includes the recruitment of 53BP1 to, and phosphorylation of H2AX at, telomeres; this process can be visualized by the presence of foci, referred to as *t*elomere-dysfunction-*i*nduced *f*oci (TIF), that represent colocalization of 53BP1 or γH2AX with shelterin components such as TRF2 or TIN2 [83, 84]. This method of monitoring telomere disruption is referred to as a TIF response [83]. By contrast, DNA double strand breaks at non-telomeric sites in the genome, such as occur following treatment with etoposide, also lead to a DNA damage response that includes recruitment of 53BP1 and phosphorylation of H2AX, however, these foci do not co-localize with telomere-associated proteins [44]. In untreated control cells grown in complete medium (FBS), 53BP1 foci rarely colocalize with TRF2 [44]. 53BP1 foci are counted and data are expressed as the percentage of cells with a specified number of foci/cell. For LNCaP cells, the cutoff is 5, as about 80% of untreated LNCaP cells have ≤5 53BP1 foci/cell [44]; for LAPC4 cells, the cutoff was 10, suggesting that these cells have a higher level of DNA damage, though at non-telomeric sites.

### Indirect immunofluorescence

The immunofluorescent staining of cells grown on glass slides was performed as described [85]. Cells were fixed with 4% paraformaldehyde, permeabilized with 0.5 % Triton X-100 and incubated at 4°C overnight with antibodies against TIN2 [85], AR (AR-N20, AR-441, Santa Cruz), 53BP1 (Abcam), γH2AX (i.e., phosphorylated-H2AX) (Upstate), or TRF2 (IMG-124A, Imgenex). After washing, cells were stained with goat-anti-rabbit-FITC and/or goat-anti-mouse-Texas Red (Molecular probes) secondary antibodies. Images of cells were acquired on an LSM-410 confocal microscope (Zeiss).

### p53 knockdown

LNCaP cells were infected with a dominant-negative p53 (GSE-22) [51] or control retroviral expression vector (pBabe), and selected with 0.5 μg/ml puromycin for 2-3 d as described in Kim et. al. [9]. Loss of p53 activity was confirmed by the absence of detectable p21 (BD Pharmingen) immunostaining in GSE-22-expressing cells treated with 10 μg/ml etoposide (Sigma) for 1 hr.

### AR knockdown

Exponentially growing LNCaP cells (1.0 −2.0 × 10^5^ cells/wellof a six-well plate) were transfected with 200 pmol of AR-siRNA (Sc-29204, Santa Cruz) or scrambled-siRNA (Santa Cruz), using Lipofectamine 2000 (Invitrogen) following the manufacturer's instructions. Cells were processed48 h later for immunofluorescence staining or Western blotting.

### Cell extracts and Western blot analysis

Cells were digested with trypsin, washed with PBS and suspended in Buffer A (50 mM Tris-HCl, pH 7.4, 250 mM NaCl, 0.1% Triton X-100, 5 mM EDTA, 50 mM NaF, and 0.1 mM Na_3_VO_4_) supplemented with protease inhibitor mixture (P-8340, Sigma) as described [86]. Cells were then subjected twice to 30 pulses of sonication with a Branson Sonifier 250 set at output control 2 and duty cycle 20, with intermittent cooling on ice. The sonicated cell extract was cleared by centrifugation in an Eppendorf centrifuge at 12,500 rpm for 10 min. For Western blot analysis, membranes were probed with antibodies against AR (AR-N20, Santa Cruz), TRF2 (IMG-124A, Imgenex), TIN2 [85], TPP1 (A303-069A, Bethyl Laboratories), Rap1 (A300-306A, Bethyl Laboratories), HIS 3 (1791, Abcam), PSA (C-19, Santa Cruz), NKX3.1 (H-50, Santa Cruz), actin (I-19, Santa Cruz) or GAPDH (AB2302, Millipore). Immunoreactive bands were developed using horseradish peroxidase-conjugated secondary antibodies and Super-Signal WestPico chemiluminescent substrate (Pierce), and visualized using X-ray film.

### RT-PCR analysis

Total RNA was prepared as described [86]. RNA was reverse transcribed using random hexamers and oligo (dT) primer and Transcriptor Reverse Transcriptase (Roche Applied Science) according to the manufacturer's instructions. PCR of cDNA was carried out using Platinum PCR SuperMix (Invitrogen). PCR primers for PSA were 5’-gcacccggagagctgtgt (forward) and 5-gatcacgcttttgttcctgat (reverse), for NKX3.1 were 5’- gtacctgtcggcccctgaacg (forward) and 5’- gctgttatac acggagaccagg (reverse), and for GAPDH were 5’-gagatccctccaaaatcaagtg (forward) and 5’ ccttccacgatac caaagttgt (reverse). Cycle parameters were 94°C for 2 min, 94°C for 30 sec, 55°C for 30 sec and 68°C for 1 min. PSA was amplified for 25 cycles, NKX3.1 for 30 cycles and GAPDH for 25 cycles.

### Chromatin immunoprecipitation (ChIP) analysis

Cells were fixed in 1% formaldehyde in PBS for 60 min at room temperature, scraped, washed with PBS, and lysed in 1% SDS, 50 mM Tris-HCl, pH 8.0, 10 mM EDTA at a density of 10^7^ cells/ml, as described by Loayza and de Lange [87]. The lysate was sonicated using a Branson Sonifier 250 under the following conditions: output control of 5.5 and duty cycle of 50% with six cycles of 20 sec with intermittent cooling on ice. Lysate (0.2 ml) was diluted with 1.2 ml Buffer (0.01% SDS, 1.1% Triton X-100, 1.2 mM EDTA, 16.7 mM Tris-HCl, pH 8.0, and 150 mM NaCl), incubated at 4°C overnight with antibody (5 μg of AR N-20; 5 μg of Rabbit IgG; 5 μg of Goat TRF2; 5 μg of Rabbit Rap1), and then antibody-bound material was precipitated with 30 μl protein-G Sepharose beads (Invitrogen) that had been pre-equilibrated with 30 μg bovine serum albumin (BSA) and 5 μg sheared *Escherichia coli* DNA for 30 min at 4°C. In order to isolate DNA from the ChIP pellet (ChIP DNA), cross-linking was reversed at 65°C for 4 h, treated with RNAase A and proteinase K at 37 °C, and extracted with 0.5 ml phenol/chloroform/iso- amylalcohol. In order to probe for telomere DNA, equal amounts of ChIP DNA were applied as dots to a nylon membrane (Invitrogen) using a dot-blot apparatus (Bio-Rad). The membrane was then hybridized with a digoxigenin (DIG)-labeled telomere DNA (gggtaa)_10_ probe at 42°C overnight. DIG labeling and detection were performed using the DIG Oligonucleotide 3-End Labeling and Detection Kit (Roche). After capturing the signals produced by this hybridization, the membrane was stripped by washing twice with 0.2 M NaOH and 0.1% SDS for 30 min at 52°C and then re-hybridized with a DIG-labeled mutant telomeric (gcctaa)_10_ probe. In order to probe ChIP DNA for AR binding sites in the PSA gene, ChIP DNA was subjected to PCR using primers for PSA ARE III: 5’- cttctagggtgaccagagcag (forward) and 5’- gcaggcatccttg-caagatg (reverse). Cycle parameters were 94°C for 2 min, 94°C for 30 sec, 55°C for 30 sec and 68°C for 1 min for 30 cycles.

### PICh protocol to isolate telomeric chromatin

The PICh protocol was performed essentially as described by Dejardin and Kingston {[64] and http://genetics.mgh.harvard.edu/kingstonweb/labmembers1.html}. Cells were treated with 3% formaldehyde in PBS for 30 min at room temperature (RT) prior to harvesting. Nuclei were isolated by homogenization and digested with micrococcal nuclease (MN) for 30 min at 37°C. MN-treated nuclei were sonicated using a Branson Sonifier 250 (output 5.5 with 28 cycles of 15 sec/cycle) with intermittent cooling on ice to further reduce the length of chromatin fragments to ~600 bp. Fragmented chromatin was then hybridized to a biotinylated telomere DNA probe [desthiobiotin-108 carbons-5′TtAgGgTtAgGgTtAgGgTtAgGgt-3′; capital letters indicate locked nucleic acid (LNA) residues, which increase the stability of probe-chromatin interactions], or to a biotinylated scrambled DNA probe (desthiobiotin-108 carbons-5’ GaTgTgTgGaTgTggAt-GtGgAtgTgg-3’). Hybridization conditions were 25°C for 3 min, 70°C for 6 min (3 cycles), 38°C for 60 min, 60°C for 2 min, 38°C for 60 min, 60°C for 2 min, 38°C for 120 min, and 25°C final temperature, using an MJ PTC-200 PCR machine. The probes contain a long 108-carbon spacer between the immobilization tag (desthiobiotin) and the LNA probe; the spacer was designed to minimize steric hindrance [88]. These probes were synthesized by Fidelity Systems (Gaithersburg, MD) and Exiqon (Woburn, MA). The hybridized chromatin was captured onto magnetic streptavidin beads. The beads were washed extensively under stringent conditions at 42°C, and then protein was eluted using sample buffer for Western blot analysis of AR (AR antibody N-20, Santa Cruz) and shelterin components {antibodies against TRF2 (Imgenex), Rap1(Bethyl laboratories), and TIN2 [9, 44]}. Histone 3 (Abcam) signal intensity served as an indicator of sample loading.

### Fluorescence in situ hybridization (FISH) analysis of telomeres

Telomeres were visualized by fluorescence *in situ* hybridization (FISH) on metaphase spreads using a telomeric protein nucleic acid (PNA) probe, as described [89]. LNCaP cells were treated with 100 μM Casodex for 24 hr, then washed in medium without Casodex, and incubated overnight at 37°C in medium containing colcemid (0.1 μg/ml, Sigma). Cells were trypsinized and collected at 1000 × *g* (8 min). After hypotonic swelling in 0.075 mM KCl for 25 min at 37^o^ C, cells were fixed in methanol:acetic acid (3:1). Telomere FISH was performed as described [90], using a Cy3-OO-(CCCTAA)_3_-peptide nucleic acid (PNA) probe (Panagene).
